# A child's experience as a patient in a pediatric intensive care unit: what we learned through their drawings and narratives

**DOI:** 10.1186/2197-425X-3-S1-A742

**Published:** 2015-10-01

**Authors:** M Clark, M Carleton, A Kelleher, N Noviski

**Affiliations:** Massachusetts General Hospital, Pediatric Critical Care Medicine, Boston, United States; Massachusetts General Hospital, Cancer Center, Boston, United States

## Introduction

Children admitted to a Pediatric Intensive Care Unit (PICU) have physical and emotional experiences that differ from other pediatric patients due to their complicated needs and severity of their conditions. This is the first study to examine a child's experience as a patient in the PICU through their drawings.

## Objectives

To gain insight into what it is like to be a patient in a PICU through children's drawings and narratives of their experience.

## Methods

Prospective study, single center study in a 14-bed pediatric ICU in a tertiary care, academic-affiliated hospital. Patients were asked to identify the best and worst things about their PICU experience. Patients were then asked to “draw what it is like to be a patient in the PICU” and to describe the drawing so details of the drawing were interpreted as intended.

## Results

Forty patients (6-17 years old) were enrolled. The most common theme in 88% (n=35) of drawings was patients depicting themselves in their drawings. Of those 35 patients, 14 depicted themselves with a smile on their face; 15 drew a frown, tears or an angry expression on their face; 6 patients drew themselves as expressionless. Patient monitors were depicted in 53% (n=21) of drawings. Four out of these 21 drawings referred to the monitor alarms as “loud”, “disruptive”, “annoying” or “kept me from sleeping”. In 50% (n=20) of the drawings, IVs were represented. In all 20 of the drawings IVs were represented as negative and associated with pain and fear. In 35% (n=14) of the drawings, nurses were depicted. Twelve patients reflected their experience with the nurses as positive, 2 patients reported negative experiences. Television was reflected in the drawings of 13 patients (33%) and patients reported watching television was a “distraction” from feelings of “pain”, “fear”, “boredom” or “feeling lonely.” When asked to choose “the best thing about your PICU experience”, the PICU staff (physicians, nurses, food service provider) was the most common response (25%). The response most frequently voiced by patients (25%) when asked about “the worst thing about your PICU experience” was having an IV.

## Conclusions

Drawing as a means of communication enables patients admitted to a PICU to disclose sensitive, descriptive information about their experience as a patient that would not have been known otherwise.
